# Apatinib Plus Toripalimab (Anti-PD1 Therapy) as Second-Line Therapy in Patients With Advanced Gastric or Esophagogastric Junction Cancer: Results From a Randomized, Open-Label Phase II Study

**DOI:** 10.1093/oncolo/oyae005

**Published:** 2024-02-16

**Authors:** Qing Wei, Xiaoqing Xu, Jingjing Li, Chang Wang, Weijun Chen, Yanru Xie, Cong Luo, Lei Chen, Jiadong Chu, Wei Wu, Zhe Han, Yanlian Yang, Zhiyuan Hu, Qi Xu, Jieer Ying

**Affiliations:** Department of Hepato-Pancreato-Biliary and Gastric Medical Oncology, Zhejiang Cancer Hospital, Hangzhou Institute of Medicine (HIM), Chinese Academy of Sciences, Hangzhou, People’s Republic of China; Key Laboratory of Prevention, Diagnosis and Therapy of Upper Gastrointestinal Cancer of Zhejiang Province, Hangzhou, People’s Republic of China; Department of Hepato-Pancreato-Biliary and Gastric Medical Oncology, Zhejiang Cancer Hospital, Hangzhou Institute of Medicine (HIM), Chinese Academy of Sciences, Hangzhou, People’s Republic of China; Department of Hepato-Pancreato-Biliary and Gastric Medical Oncology, Zhejiang Cancer Hospital, Hangzhou Institute of Medicine (HIM), Chinese Academy of Sciences, Hangzhou, People’s Republic of China; Cancer Center, The First Hospital of Jilin University, Changchun, People’s Republic of China; Department of Radiotherapy, Taizhou Central Hospital, Taizhou, Zhejiang, People’s Republic of China; Department of Medical Oncology, Lishui Municipal Central Hospital, Lishui, Zhejiang, People’s Republic of China; Department of Hepato-Pancreato-Biliary and Gastric Medical Oncology, Zhejiang Cancer Hospital, Hangzhou Institute of Medicine (HIM), Chinese Academy of Sciences, Hangzhou, People’s Republic of China; Department of Hepato-Pancreato-Biliary and Gastric Medical Oncology, Zhejiang Cancer Hospital, Hangzhou Institute of Medicine (HIM), Chinese Academy of Sciences, Hangzhou, People’s Republic of China; Department of Clinical Research, Zhejiang Cancer Hospital, Hangzhou Institute of Medicine (HIM), Chinese Academy of Sciences, Hangzhou, People’s Republic of China; Department of Pathology, Zhejiang Cancer Hospital, Hangzhou Institute of Medicine (HIM), Chinese Academy of Sciences, Hangzhou, People’s Republic of China; Radiology Department, Zhejiang Cancer Hospital, Hangzhou Institute of Medicine (HIM), Chinese Academy of Sciences, Hangzhou, People’s Republic of China; Nanopep Biotech. Corp., Beijing, People’s Republic of China; Nanopep Biotech. Corp., Beijing, People’s Republic of China; Department of Hepato-Pancreato-Biliary and Gastric Medical Oncology, Zhejiang Cancer Hospital, Hangzhou Institute of Medicine (HIM), Chinese Academy of Sciences, Hangzhou, People’s Republic of China; Department of Hepato-Pancreato-Biliary and Gastric Medical Oncology, Zhejiang Cancer Hospital, Hangzhou Institute of Medicine (HIM), Chinese Academy of Sciences, Hangzhou, People’s Republic of China; Key Laboratory of Prevention, Diagnosis and Therapy of Upper Gastrointestinal Cancer of Zhejiang Province, Hangzhou, People’s Republic of China

**Keywords:** gastric or esophagogastric junction cancer, second-line therapy, immunotherapy, apatinib

## Abstract

**Background:**

This study aimed to assess the activity of apatinib plus toripalimab in the second line for patients with advanced gastric or esophagogastric junction cancer (GC/EGJC).

**Methods:**

In this open-label, phase II, randomized trial, patients with advanced GC/EGJC who progressed after first-line chemotherapy were enrolled and received 250 mg apatinib per day plus 240 mg toripalimab on day 1 per 3 weeks (arm A) or physician’s choice of chemotherapy (PC, arm B). The primary endpoint of this study was the 1-year survival rate. Progression-free survival (PFS), overall survival (OS), overall response rate (ORR), and safety were assessed as secondary endpoints.

**Results:**

Twenty-five patients received apatinib plus toripalimab while 26 were enrolled in arm B. The 1-year survival rates of the 2 groups were 43.3% and 42.3%, respectively (*P* = .903). The PFS was 2.77 versus 2.33 months (*P* = .660). The OS was 8.30 versus 9.88 months (*P* = .539). An objective response was reported in 20.0% of patients in arm A compared to 26.9% in arm B (*P* = .368), respectively. A total of 6 (24.0%) patients experienced adverse events of grade ≥ 3 in arm A, while 9 (34.6%) patients suffered from adverse events of grade ≥ 3 in arm B. No drug-related deaths occurred in either group.

**Conclusion:**

Toripalimab plus apatinib treatment in second-line therapy of advanced GC/EGJC showed manageable toxicity but did not improve clinical outcomes relative to PC treatment (ClinicalTrials.gov Identifier: NCT04190745).

## Teaser

Reporting results of clinical trial NCT04190745, this article assesses the activity of apatinib plus toripalimab in the second line for patients with advanced gastric or esophagogastric junction cancer.

**Lessons Learned**

- The combination of apatinib and toripalimab was well tolerated as second-line therapy in patients with metastatic gastric or esophagogastric junction cancer.
- The primary endpoint was not met, as this combination of agents did not significantly improve 1-year survival rate.
- Although the results of this clinical trial were negative, the study provides useful learnings to guide the design of future clinical trials.

## Discussion

Apatinib, a selective tyrosine kinase inhibitor for vascular endothelial growth factor receptor 2 (VEGFR2), is commonly used as a third-line treatment for patients with GC and has been authorized by the China Food and Drug Administration. Clinical studies have shown that combining immunotherapy with apatinib yields favorable antitumor activity and manageable safety. In a phase I study involving patients with advanced cancer, a dose of 250 mg for apatinib in combination with sintilimab was recommended. Interestingly, a minority of patients with advanced GC who had received a combination of reduced dose of apatinib and anti-PD-1 antibody showed anti-cancer activity.

**Figure 1.**
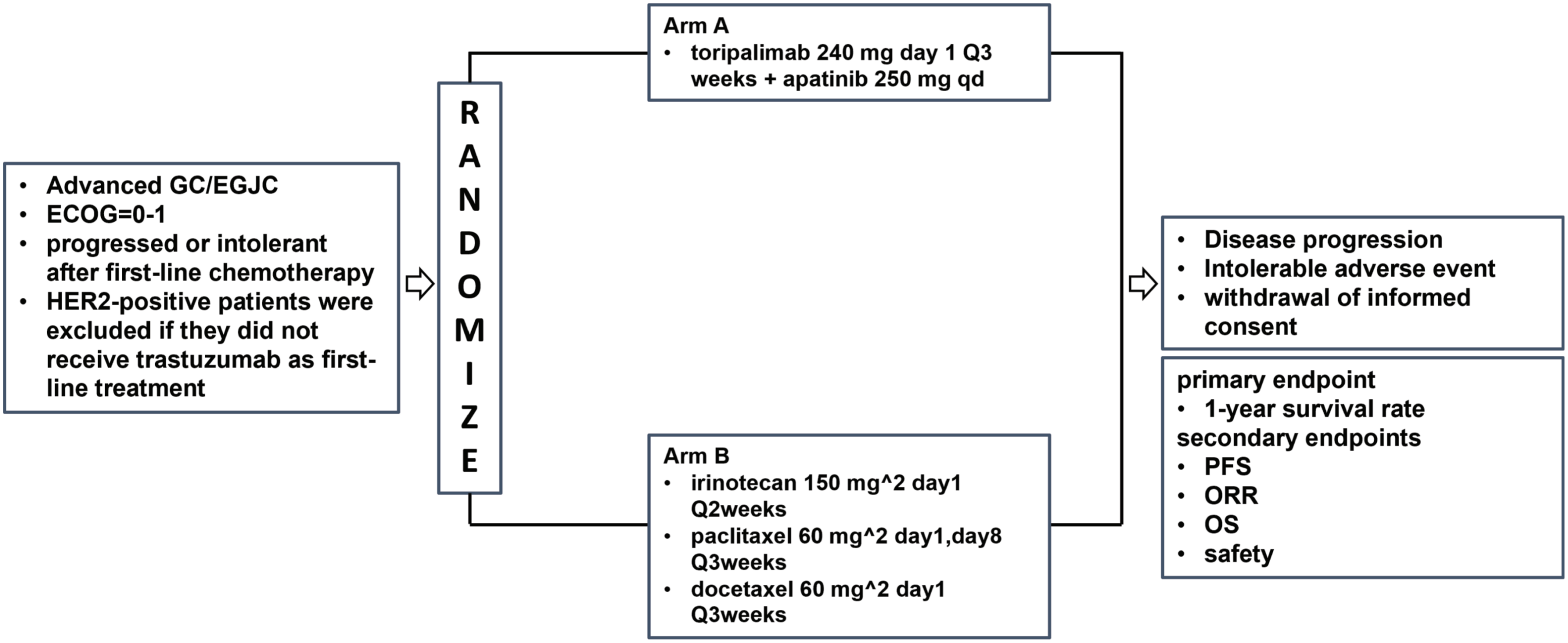
Study schema. Abbreviations: GC/EGJC: gastric or esophagogastric junction cancer; ECOG: Eastern Cooperative Oncology Group performance; PFS: progression-free survival; ORR, overall response rate; OS, overall survival.

The synergistic effect of anti-VEGFR and anti-PD-1/PD-L1 treatment in metastatic GC/EGJC has not been compared to standard chemotherapy in a randomized trial. This study is a phase II trial that is open-label, randomized, and recruiting stage IV patients aged 18-75 years from 4 centers in China. In the combination group, toripalimab was administered intravenously while apatinib was administered orally. The physician’s choice of chemotherapy (PC) group received irinotecan monotherapy, paclitaxel monotherapy, or docetaxel monotherapy. All dosages were determined based on actual body surface area (Fig. 1).

This phase II trial was designed with an α of 0.10 and β of 0.20, resulting in a planned enrollment of 58 patients per group calculated through statistical analysis. However, due to recruitment challenges resulting from COVID-19 pandemic restrictions, the experiment was terminated. In the end, for the efficacy analysis in this study, a total of 51 patients were enrolled between November 21, 2019, and July 24, 2021. The baseline characteristics of patients in both groups were well-balanced. The median follow-up duration was 16.2 months. The 1-year survival rates for the toripalimab plus apatinib group and PC group were 43.3% and 42.3%, respectively (*P* = .903). At 18 months, the overall survival (OS) rates were 31.0% versus 23.9%, and at 24 months, they were 31.0% versus 8.0%. Among the adverse events, 24% of patients in the combination group experienced grade ≥ 3 events, while 36% of patients in the PC group had grade ≥ 3 events. In the combination group, anemia (3/25, 12%) and elevated γ-glutamyl transferase levels (2/26, 8%) were the most common grade ≥ 3 adverse events. The most prevalent grade ≥ 3 adverse event in the PC group was decreased neutrophil count (7/26, 26.92%). It is noteworthy that no drug-related deaths occurred in either group. Our findings indicate that the dual application of toripalimab and apatinib does not exhibit noteworthy antitumor efficacy in the second-line treatment.

**Table:** 

Trial Information	
Disease	Gastric or esophagogastric junction cancer (GC/EGJC)
Stage of disease/treatment	Advanced/metastatic
Prior therapy	One prior regimen
Type of study	Open-label, phase II, randomized trial
Primary endpoint	1-year survival rate
Secondary endpoints	Progression-free survival (PFS), OS, overall response rate (ORR)
Investigator’s analysis	Correlative endpoints were not met but clinical activity wae observed. The level of activity did not meet the planned endpoint.

## Additional Details of Endpoints or Study Design

This study is an open-label, randomized, phase II trial recruiting stage IV patients aged 18-75 years from 4 centers in China. Eligible patients had a histologically confirmed diagnosis of GC/EGJC cancer with an Eastern Cooperative Oncology Group (ECOG) performance status of 0-1. These patients progressed or intolerant after first-line chemotherapy and had a minimum of one lesion that was measurable, as classified in the efficacy evaluation criteria for Solid Tumors (RECIST) version 1.1. HER2-positive patients were excluded from this analysis if they did not receive trastuzumab as first-line treatment. Other inclusion criteria were proper organic and bone-marrow functions for 28 days before dosing on day 1 of cycle 1. Patients with pre-existing autoimmune disease, active primary bleeding, brain metastases, or previous treatment with VEGFR inhibitors (eg, sorafenib, sunitinib, regorafenib, and levatinib) were excluded. This research was performed according to the protocol approved by the Human Research Ethics Committee (No. IRB-2019-155). The informed consent was given by all patients prior to their participation in the study. Trial registration number: NCT04190745.

**Table:** 

Drug Information (Multi-Arm Trial)		
Arm	Arm A	Arm B
Generic/working name	Toripalimab plus apatinib	Irinotecan or paclitaxel or docetaxel
Company name drug type	Shanghai Junshi Bioscience Co., Ltd., monoclonal antibody Jiangsu Hengrui Pharmaceuticals Co., Ltd., small molecule	Commercially available
Drug class	PD-1 antibody/selective VEGFR-2 tyrosine kinase inhibitor	Chemotherapy
Dose	Toripalimab: 240 mg on day 1 per 3 weeks; Apatinib: 250 mg/day for 21-day cycles	Irinotecan: 150 mg/m^2^ of body surface area per dose, or paclitaxel: 80 mg/m^2^ of body surface area per dose, or docetaxel: 60 mg/m^2^ of body surface area per dose
Route	IV	IV
Schedule of administration	Per 3 weeks	Irinotecan: 150 mg/m^2^ of body surface area administered per 2 weeks, or paclitaxel monotherapy, with a dosage of 80 mg/m^2^ of body surface area, administered on days 1 and 8 per 3 weeks, or docetaxel mono-therapy, with a dosage of 60 mg/m^2^ administered on day 1 per 3 weeks

**Table:** 

Patient Characteristics: PC	
Number of patients, male	19
Number of patients, female	7
Stage	IV
Age, median (range)	59.8 (27-77) years
Number of prior systemic therapies	1
Performance status: ECOG	
0	11
1	15
2	0
3	0
4	0
Cancer types or histologic subtypes	Intestinal, 6; diffuse, 18; mixed, 2

**Table:** 

Patient Characteristics: Apatinib Plus Toripalimab	
Number of patients, male	17
Number of patients, female	8
Stage	IV
Age, median (range)	59.2 (27-75) years
Number of prior systemic therapies	1
Performance status: ECOG	
0	10
1	15
2	0
3	0
4	0
Cancer types or histologic subtypes	Intestinal, 6; diffuse, 15; mixed, 4

**Figure 2.**
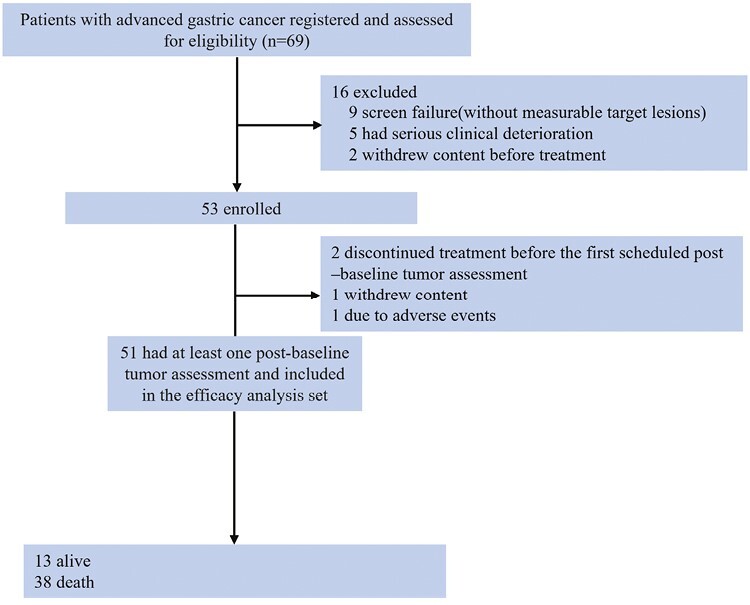
Flowchart.

See also Figure 2 and Table 1.

**Table 1.** Patient clinicopathological characteristics.

	Apatinib plus toripalimab	Physician’s choice of chemotherapy	*P* value
Age (years)	59.2	59.8	
Median age, years			
<65	20 (80.0%)	17 (65.4%)	.197
≥65	5 (20.0%)	9 (34.6%)	
Sex			
Male	17 (68.0%)	19 (73.1%)	.464
Female	8 (32.0%)	7 (26.9%)	
ECOG performance status			
0	10 (40.0%)	11 (42.3%)	.547
1	15 (60.0%)	15 (57.7%)	
Histology subtype (Lauren classification)			
Intestinal	6 (24.0%)	6 (23.1%)	.631
Diffuse	15 (60.0%)	18 (69.2%)	
Mixed	4 (16.0%)	2 (7.7%)	
Histological differentiation			
Low	15 (60.0%)	12 (46.2%)	.239
Moderate-high	10 (40.0%)	14 (53.8%)	
Number of organs with metastases			
1	10 (40.0%)	10 (38.5%)	.569
≥2	15 (60.0%)	16 (61.5%)	
Peritoneal metastases			
Yes	5 (20.0%)	8 (30.8%)	.378
No	20 (80.0%)	18 (69.2%)	
Ascites			
Asymptomatic	3 (12.0%)	5 (19.2%)	.374
None	22 (88.0%)	21 (80.8%)	
Liver metastases			
Yes	4 (16.0%)	5 (19.2%)	.526
No	21 (84.0%)	21 (80.8%)	
Previous therapies			
Fluorouracil-based doublet	23 (92.0%)	24 (92.3%)	.68
Paclitaxel-based doublet	2 (8.0%)	2 (7.7%)	
HER2			
Positive	2 (8.0%)	3 (11.5%)	.519
Negative	23 (92.0%)	23 (88.5%)	
MSI-H			
Positive	1 (4.0%)	1 (3.8%)	.745
Negative	24 (96.0%)	25 (96.2%)	
EBV			
Positive	2 (8.0%)	2 (7.7%)	.68
Negative	23 (92.0%)	24 (92.3%)	

**Table:** 

Primary Assessment Method: Arm A, Apatinib Plus Toripalimab, 1-Year Survival Rate	
Number of patients screened	34
Number of patients enrolled	26
Number of patients evaluable for toxicity	25
Number of patients evaluated for efficacy	25
Evaluation method	RECIST 1.1
Response assessment, CR	0 (0%)
Response assessment, PR	5 (20%)
Response assessment, SD	12 (48%)
Response assessment, PD	8 (32%)
Median duration assessments, PFS	2.77 months (CI: 2.10-3.44)
Median duration assessments, TTP	2.25 months (CI: 2.01-3.02)
Median duration assessments, OS	8.30 months (CI: 5.35-13.65)
Duration of treatment	2.77 months (CI: 2.10-3.44)

## Outcome Notes

**Figure 3.**
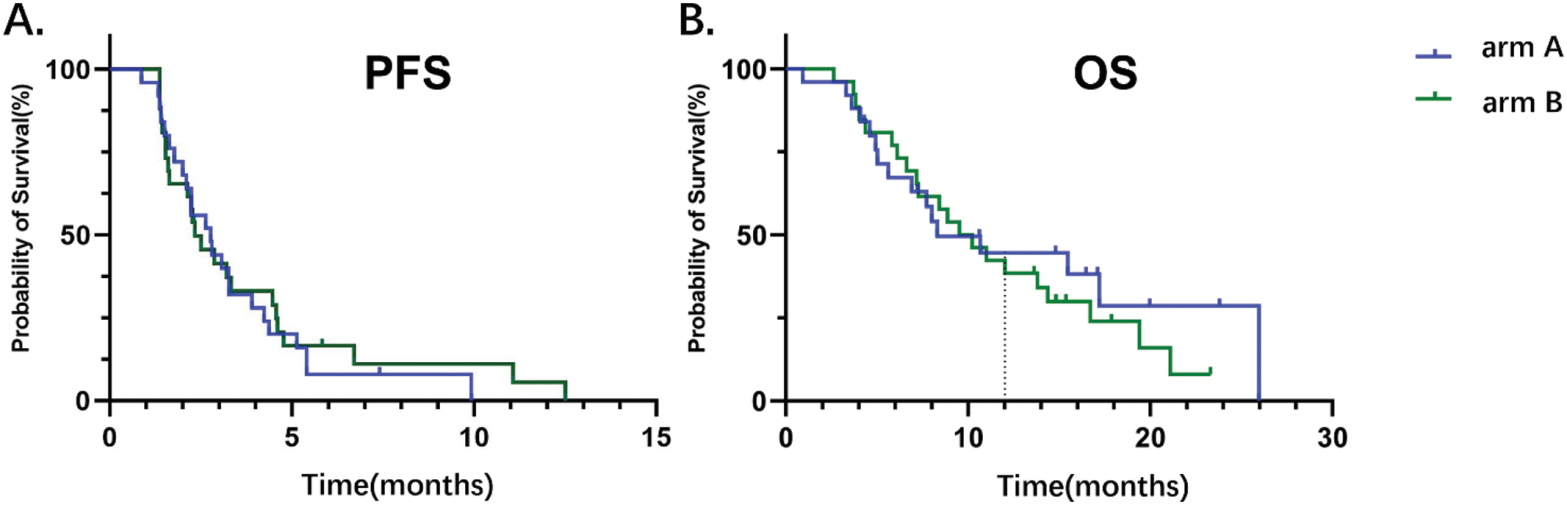
PFS and OS. arm A: apatinib and toripalimab; arm B: physician’s choice of chemotherapy. Abbreviations: PFS, progression free survival; OS,overall survival.

**Figure 4.**
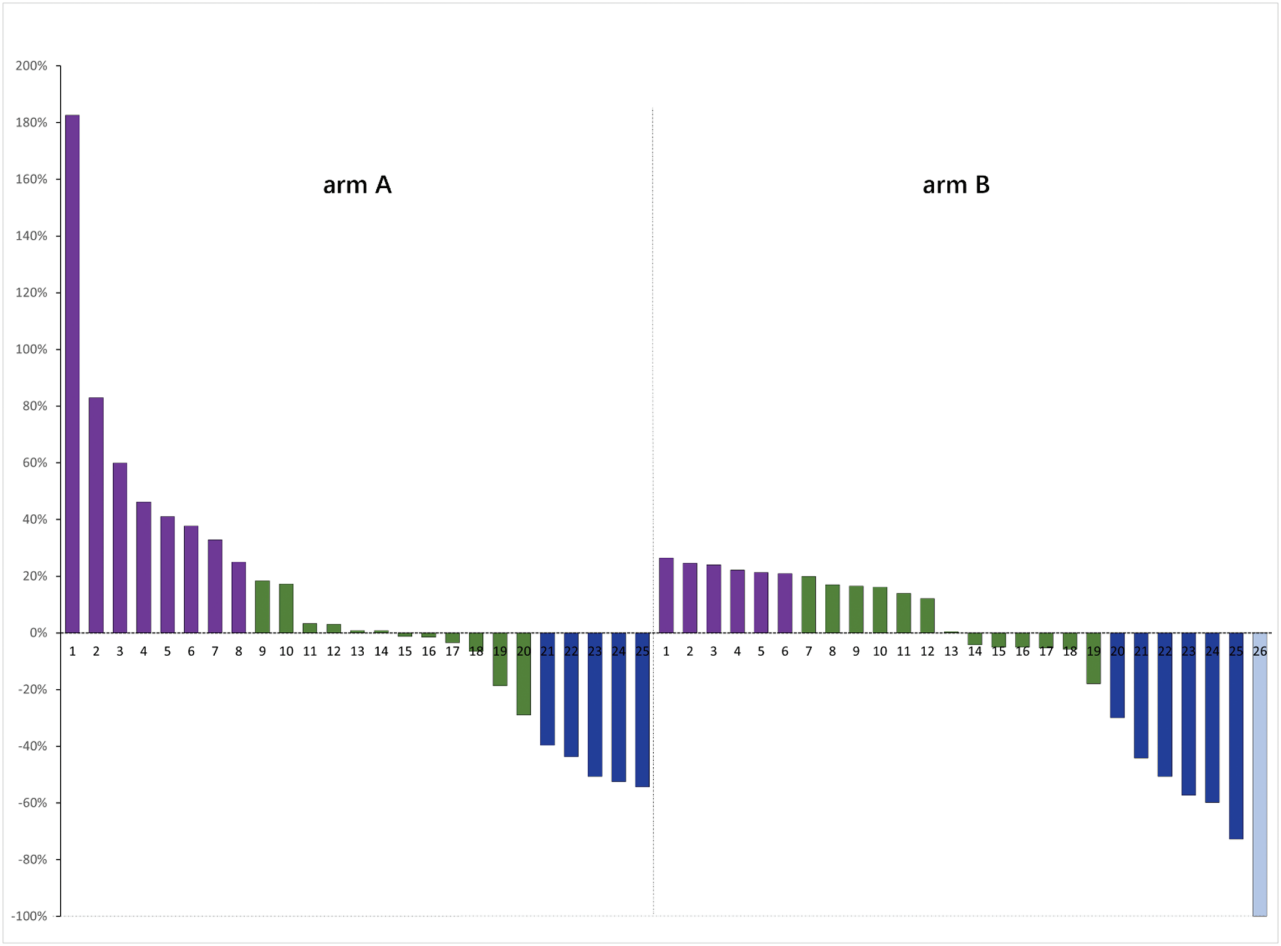
Optimal percentage change in the sum of diameters of target lesions from baseline to time on treatment. arm A: apatinib and toripalimab; arm B: physician’s choice of chemotherapy.

**Figure 5.**
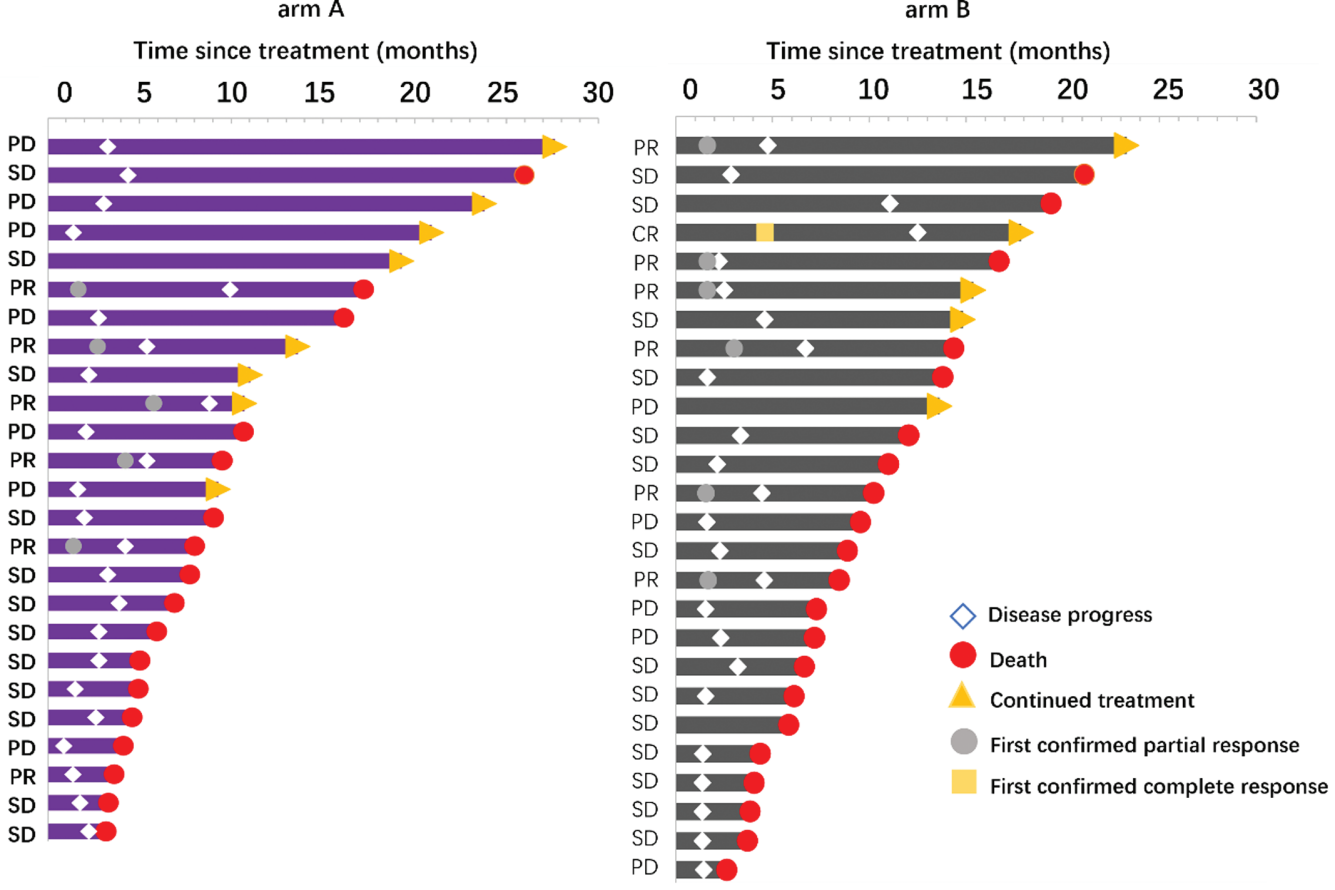
Swimmer plot of survival of the 2 groups. arm A: apatinib and toripalimab; arm B: physician’s choice of chemotherapy.

**Figure 6.**
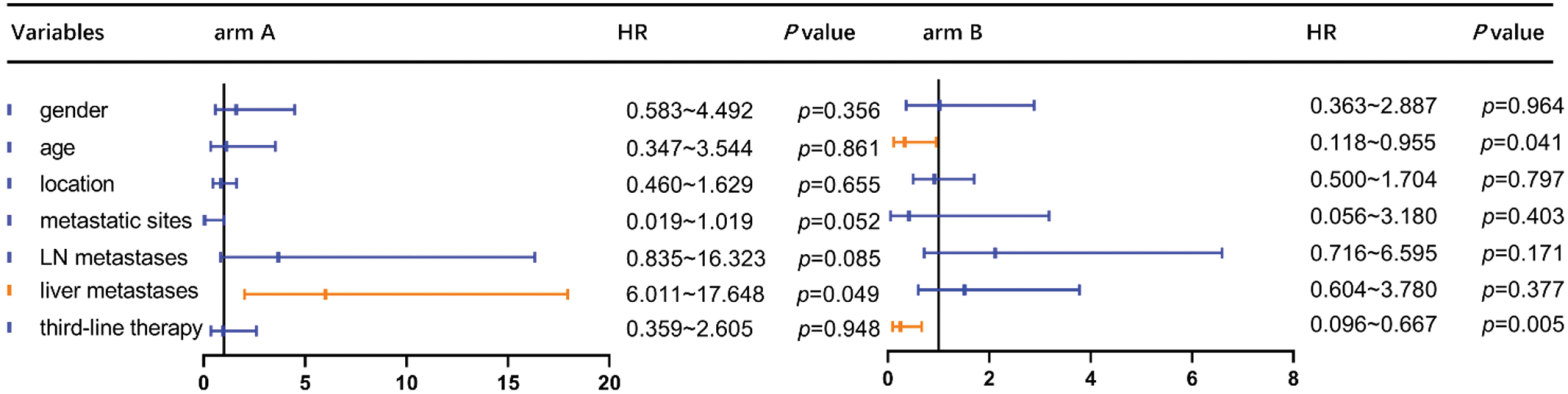
Univariate Cox regression analysis for OS. OS, overall survival. arm A: apatinib and toripalimab; arm B:physician’s choice of chemotherapy.

One-year survival rate in arm A was 43.3%. The OS of patients receiving apatinib plus toripalimab versus PC group was 8.30 versus 9.88 months (HR = 1.221; 95% CI: 0.641-2.328; *P* = .539). The PFS of patients receiving apatinib plus toripalimab group versus PC group was 2.77 versus 2.33 months (HR = 0.885; 95% CI: 0.502-1.560; *P* = .660; Fig. 3). Figure 4 shows the change in tumor burden from baseline. An objective response was reported in 20% of patients in the arm A compared to 23% in arm B (*P* = .368), respectively (Fig. 5). Eleven (44%) responses occurred in the apatinib plus toripalimab group, whereas 13 (50.0%) responses occurred in the PC group (Fig. 5). Older age (HR = 0.366, 95% CI: 0.118-0.955; *P* = .041) and third-line therapy (HR = 0.253, 95% CI: 0.096-0.667; *P* = .005) were beneficial factors for patients in the PC group, whereas liver metastasis was a detrimental factor in the Combo group (HR = 0.318, 95% CI: 0.102-0.993; *P* = .049). In the Combo group, 48% of patients received third-line therapy. In the PC group, 15/26 (57.7%) patients received third-line therapy, and 10/15 (66.7%) patients received IO treatment (Fig. 6).

**Table:** 

Primary Assessment Method: Arm B, PC, and 1-Year Survival Rate	
Number of patients screened	35
Number of patients enrolled	27
Number of patients evaluable for toxicity	26
Number of patients evaluated for efficacy	26
Evaluation method	RECIST 1.1
Response assessment, CR	1 (3.8%)
Response assessment, PR	6 (23.1%)
Response assessment, SD	14 (53.8%)
Response assessment, PD	5 (19.2%)
Median duration assessments, PFS	2.33 months (CI: 1.57-3.09)
Median duration assessments, TTP	1.97 months (CI: 1.52-2.83)
Median duration assessments, OS	9.88 months (CI: 6.32-12.74)
Duration of treatment	2.33 months (CI: 1.57-3.09)

## Outcome Notes

One-year survival rate in arm B was 42.3%. Drug-related adverse events were experienced by 50/51 (98.0%) of patients (Table 2). Grade ≥ 3 drug-related adverse events occurred in 6/25 (24%) patients in arm A, as compared to 9/25 (36%) patients in the arm B. Common grade ≥ 3 adverse events were anemia (3/25, 12%) and γ-glutamyl transferase increased (2/26, 8%) in arm A. Decreased neutrophil count (7/26, 26.92%) was the most common adverse event in the PC group.

**Table 2.** Drug-related adverse events.

Preferred term	Apatinib plus toripalimab (*N* = 25)				Physician’s choice of chemotherapy(*N* = 26)			
	Any grade	Grades 1-2	Grade 3	Grade 4	Any grade	Grades 1-2	Grade 3	Grade 4
Any adverse events	24 (96.00%)	18 (72.00%)	3 (12.00%)	3 (12.00%)	26 (100%)	17 (65.38%)	7 (26.92%)	2 (7.69%)
Anemia	20 (80.00%)	17 (68.00%)	3 (12.00%)	0 (0.00%)	26 (100%)	25 (96.15%)	1 (3.85%)	0 (0.00%)
Hypoalbuminemia	19 (76.00%)	19 (76.00%)	0 (0.00%)	0 (0.00%)	15 (57.69%)	15 (57.69%)	0 (0.00%)	0 (0.00%)
Proteinuria	17 (68.00%)	16 (64.00%)	1 (4.00%)	0 (0.00%)	4 (15.38%)	4 (15.38%)	0 (0.00%)	0 (0.00%)
γ-Glutamyltransferase increased	13 (52.00%)	11 (44.00%)	2 (8.00%)	0 (0.00%)	11 (42.31%)	9 (34.62%)	1 (3.85%)	1 (3.85%)
Hypopotassemia	13 (52.00%)	12 (48.00%)	1 (4.00%)	0 (0.00%)	4 (15.38%)	3 (11.54%)	1 (3.85%)	0 (0.00%)
Aspartate aminotransferase increased	13 (52.00%)	13 (52.00%)	0 (0.00%)	0 (0.00%)	6 (23.08%)	6 (23.08%)	0 (0.00%)	0 (0.00%)
Alanine aminotransferase increased	12 (48.00%)	12 (48.00%)	0 (0.00%)	0 (0.00%)	8 (30.77%)	7 (26.92%)	1 (3.85%)	0 (0.00%)
Platelet count decreased	10 (40.00%)	10 (40.00%)	0 (0.00%)	0 (0.00%)	8 (30.77%)	8 (30.77%)	0 (0.00%)	0 (0.00%)
Hyponatremia	9 (36.00%)	7 (28.00%)	0 (0.00%)	0 (0.00%)	3 (11.54%)	3 (11.54%)	0 (0.00%)	0 (0.00%)
Neutrophil count decreased	7 (28.00%)	7 (28.00%)	0 (0.00%)	0 (0.00%)	24 (92.31%)	17 (65.38%)	5 (19.23%)	2 (7.69%)
Conjugated bilirubin increased	7 (26.92%)	6 (23.08%)	0 (0.00%)	1 (3.85%)	1 (3.85%)	0 (0.00%)	1 (3.85%)	0 (0.00%)
Unconjugated bilirubin increased	6 (23.08%)	6 (23.08%)	0 (0.00%)	0 (0.00%)	8 (30.77%)	7 (26.92%)	1 (3.85%)	0 (0.00%)
Fatigue	6 (23.08%)	6 (23.08%)	0 (0.00%)	0 (0.00%)	8 (30.77%)	8 (30.77%)	0 (0%)	0 (0.00%)
White blood cell count decreased	6 (23.08%)	6 (23.08%)	0 (0.00%)	0 (0.00%)	21 (80.77%)	19 (73.08%)	2 (7.69%)	0 (0.00%)
Pain	6 (23.08%)	6 (23.08%)	0 (0.00%)	0 (0.00%)	4 (15.38%)	4 (15.38%)	0 (0.00%)	0 (0.00%)
Total bilirubin increased	5 (19.23%)	4 (15.38%)	1 (3.85%)	0 (0.00%)	1 (3.85%)	1 (3.85%)	0 (0.00%)	0 (0.00%)
Urinary tract infection	4 (15.38%)	4 (15.38%)	0 (0.00%)	0 (0.00%)	6 (23.08%)	6 (23.08%)	0 (0.00%)	0 (0.00%)
Hypertension	3 (11.54%)	2 (7.69%)	1 (3.85%)	0 (0.00%)	0 (0%)	0 (0.00%)	0 (0.00%)	0 (0.00%)
Creatinine increased	3 (11.54%)	3 (11.54%)	0 (0.00%)	0 (0.00%)	4 (15.38%)	3 (11.54%)	1 (3.85%)	0 (0.00%)
Vomiting	2 (7.69%)	2 (7.69%)	0 (0.00%)	0 (0.00%)	1 (3.85%)	1 (3.85%)	0 (0.00%)	0 (0.00%)

## Pharmacokinetics and Pharmacodynamics

Circulating tumor cells (CTCs) were isolated and counted using the TumorFisher method (Nanopep Biotech). PD-L1 expression on CTCs was categorized based on the mean fluorescence intensity. The PD-L1 CPS evaluation was performed on 8 patients in arm A. Interestingly, the PD-L1 CPS for archived tissue did not correlate with the number of PD-L1-positive CTCs (Table 3). Additionally, the PD-L1 CPS was not related to the image evaluation. Baseline PD-L1 CPS might therefore not be an ideal biomarker for identifying patients who can benefit from anti-PD-1 therapy. Moreover, baseline PD-L1-positive CTCs counts appeared to be associated with efficacy.

**Table 3.** Dynamic changes in PD-L1-positive CTCs following apatinib plus toripalimab therapy.

No.	Treatment response	CPS	Before		After	
			CTC count	PD-L1-positive cells count	CTC count	PD-L1-positive cells count
No. 1	PR	1	20	5	14	2
No. 2		15	33	5	24	0
No. 3		2	13	1	12	0
No. 4	SD	3	21	1	11	0
No. 5		20	1	0	2	1
No. 6		20	5	0	1	0
No. 7	PD	<1	7	2	23	1
No. 8		40	6	0	6	0

**Figure 7.**
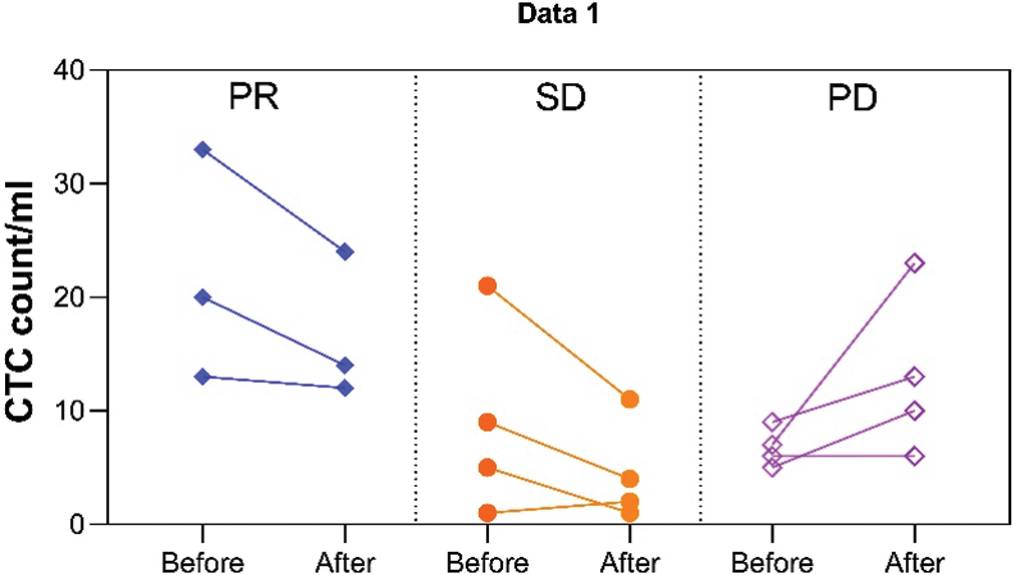
Dynamic changes in CTCs following apatinib plus toripalimab therapy. Abbreviations: CTC, circulating tumor cell; PR, partial response; SD, stable disease; PD, progressive disease.

Next, we evaluated whether CTCs count could predict treatment efficacy. A total of 11 patients in arm A underwent CTC detection dynamically before and after 2 cycles of treatment. The median number of baseline CTCs was 13. In patients whose image evaluations showed PRs, the CTC number decreased (*P* = .109), whereas in patients whose image evaluations showed progressive diseases (PDs), the CTC number increased (*P* = .317) which were tested by Wilcoxon Signed Rank Tests (Fig. 7). Due to the relatively small sample size and the potential for statistical bias, it can only serve as a reference, but we can still observe the trends.

## Assessment, Analysis, and Discussion

**Table:** 

Completion	Study Terminated Prior to Completion
Investigator’s Assessment	Correlative endpoints were not met but clinical activity was observed. The level of activity did not meet the planned endpoint.

Monotherapy (anti-angiogenic therapy or immunotherapy) has demonstrated limited efficacy in treating advanced GC/EGJC. VEGF inhibition and PD-1 blockade have been investigated in numerous clinical trials, most combinations have yielded favorable outcomes.^1,2^ However, few studies have compared this regimen with standard chemotherapy. As far as is currently known, this is the largest randomized study to assess the activity of the combination of apatinib and an immune checkpoint inhibitor in GC/EGJC. Standard second-line treatment involves paclitaxel, docetaxel, or irinotecan monotherapy.^3,4^ The median OS in the PC group was in line with previously observed.^3,4^ Although ramucirumab plus paclitaxel significantly improved the OS significantly in patients with second-line advanced gastric cancer compared to paclitaxel alone, and it is currently the global standard-of-care second-line therapy for patients with advanced gastric cancer and good performance status,^5,6^ ramucirumab was not available in Chinese mainland during this study, prompting us to choose mono-chemotherapy for the control group. Adverse events in both groups were consistent with our expectations. The lower number of grade ≥ 3 adverse events in the toripalimab plus apatinib group suggested favorable safety profiles and potentially an alternative treatment option.

In recent years, chemotherapy combined with immunotherapy has proven to be the standard first-line therapy.^7^ However, data from CheckMate-649, KEYNOTE-062, and KEYNOTE-059 showed no advantage in adding ICI to chemotherapy in low PD-L1-expressing GEAC tumors in the first-line setting.^7-10^ However, in later-line therapy, the PD-L1 expression assessed by IHC in the archival sample did not reflect the real-time PD-L1 status. Interventions such as chemotherapy or radiation alter PD-L1 status,^11,12^ making it essential to have an effective method to assess immediate PD-L1 status. Recent studies have demonstrated the clinical significance of CTCs,^13,14^ and PD-L1 expression on CTCs may serve as a predictor of clinical outcome after PD-1 blockade. In the study by Xu et al^15^, patients with more PD-L1-positive CTCs at baseline had more favorable clinical outcomes than those with fewer PD-L1-positive CTCs. The current study showed that PD-L1-positive CTCs were associated with the response rate and the PD-L1 IHC score. In addition, the PD-L1 CPS in archived tissue might not be a good indicator for second-line immunotherapy. Further, the detection of PD-L1 expression in CTCs could serve as a superior prognostic factor for clinical outcomes following PD-1 blockade. These findings suggest that monitoring the existence of CTCs with PD-L1 positivity prior to treatment could be a potential prognostic strategy.

This phase II trial was designed with α = 0.10 and β = 0.20. An ineffective drug combination was defined as having a 1-year OS rate at 12 months of P0 = 25% (null hypothesis), based on previously published trial results, and a promising drug combination was identified by a 1-year OS rate of P1 ≥ 45% (alternative hypothesis). Unfortunately, the experiment was terminated due to recruitment difficulties caused by COVID-19 pandemic restrictions. Many patients were unable to enroll in the study as they received treatment locally. Furthermore, during the study, immunotherapy emerged as the first-line treatment for advanced GC/EGJC, resulting in challenges for enrolling patients for immune-based second-line clinical trials. With the actual sample sizes collected (25 vs 26), the statistical power to test the 1-year OS rates between the 2 groups is estimated to be around 34%. Due to being underpowered, the study’s outcomes are inconclusive. If this study were to be conducted again, it would be worth considering if the same design is suitable. Phase II studies are frequently conducted to obtain outcomes of an innovative regimen swiftly. In future research endeavors, we could simplify the research design to a single-arm study, optimize recruitment strategies, overcome challenges related to insufficient sample sizes, and ensure data quality, similar to other studies involving immune and anti-angiogenic agents. This will enhance our ability to achieve research objectives and generate reliable results.

Patients with liver metastases receive limited benefit from immunotherapy. The liver is a common site for tumor metastasis and promotes immune tolerance in the setting of autoimmune diseases, viral infections, and organ transplantation.^16^ As a result, liver metastasis is a detrimental factor for toripalimab plus apatinib therapy. Moreover, third-line therapy is a beneficial factor for OS. In the PC group, patients who were 65 years or older experienced longer survival compared to those in the combination group, potentially due to intensive care and close monitoring. The majority of patients in the PC group received third-line immunotherapy, which may have influenced OS.

This study has some limitations. First, the combination of chemotherapy and immunotherapy is the standard first-line therapy. This was not the case when our study was designed in 2019, which posed challenges for enrolling patients, ultimately leading us to terminate the study. Second, the combination therapy did not offer a statistically significant advantage, falling below the predetermined activity threshold required to warrant phase III trials for the combination of apatinib and toripalimab. Third, we had no data on tissue PD-L1 expression due to the difficulty of rebiopsy. Last, it is necessary to explore the genomic mechanisms responsible for the prolonged survival observed in a small proportion of patients.

In summary, while the study did not achieve its intended goal of prolonging OS, the clinical trial’s design and informative research data still provide valuable insights.

## Data Availability

The findings of this study are available on request from the corresponding author.

## Notes

Principal Investigator: Jieer Ying
